# Movement behavior of a solitary large carnivore within a hotspot of human-wildlife conflicts in India

**DOI:** 10.1038/s41598-021-83262-5

**Published:** 2021-02-16

**Authors:** Dipanjan Naha, Suraj Kumar Dash, Caitlin Kupferman, James C. Beasley, Sambandam Sathyakumar

**Affiliations:** 1 Department Endangered Species Management, Wildlife Institute of India, Chandrabani, Dehradun, Uttarakhand India; 2 Savannah Research Ecology Laboratory, University of Georgia, Athens, GA USA

**Keywords:** Ecology, Zoology

## Abstract

With a rise in human induced changes to natural habitats, large predators are forced to share space and resources with people to coexist within multiple-use landscapes. Within such shared landscapes, co-occurrence of humans and predators often leads to human-carnivore conflicts and pose a substantial challenge for biodiversity conservation. To better elucidate large carnivore space use within a hotspot of human-wildlife conflicts, we used GPS data for leopards (N = 6) to identify behavioral states and document spatial patterns of resource selection in response to season and human activity periods within a fragmented landscape of North Bengal, eastern India. We identified two major behavioral states (i.e. resting and travelling). From the resource selection models, we found leopards selected habitats with dense to moderate vegetation cover and proximity to water while resting and travelling within the landscape. During the dry season, when risk of human-leopard conflicts is highest, leopards selected tea plantations, forest patches but avoided protected areas. These results suggest a potential for increase in human-carnivore conflicts and a strategy to conserve large predators within multiple-use landscapes of South Asia.

## Introduction

Large carnivores are recognized as flagship species for conservation programs globally^1,2^. As apex predators, they regulate primary consumers (i.e. herbivores) both directly and indirectly, affecting consumption of plant biomass with cascading effects on the entire ecosystem^1^. Although protected areas have been established globally to conserve endangered species, large carnivores often are forced to occupy human dominated areas beyond protected reserve boundaries to meet their reproductive and dietary requirements. Such shared landscapes represent a substantive proportion of remaining geographic distribution of carnivores worldwide^3^. However, co-occurrence of large carnivores and humans poses a conservation challenge through a combination of predation on livestock and attacks on humans, leading to human-carnivore conflicts^4,5^. Increased costs of co-existence due to recurring damage from predator attacks leads to retaliatory killings and local extinctions of large carnivores, threatening overall biodiversity of an ecosystem^1^. Hence, strategies aimed at promoting human-predator coexistence within shared landscapes has been the major focus of carnivore conservation programs^6,7^.

In spite of the threats posed due to humans, large carnivore populations can persist within close proximity of people and show behavioral adaptions to survive within human dominated landscapes^8^. Availability of domestic prey, anthropogenic food sources, and refuge from other sympatric competitors act as major attractants for large carnivores within such shared landscapes^9–11^. In areas where risk of persecution is high, large carnivores change their activity, movement, habitat use, and exhibit spatial avoidance of humans^12–14^. However, in areas where risk of anthropogenic mortality is low, human activity and anthropogenic alterations to the landscape impact carnivore behavior^15^.

For predators occupying shared landscapes, risk of encountering humans is generally higher during the day compared to night. Thus, several studies have reported increased nocturnal behavior of large carnivores in areas frequented by humans^16–20^. Such “temporal partitioning” behavior can provide carnivores access to risky yet important foraging areas^13,20^, especially at times when humans are generally inactive. Adaptation to human activity periods have allowed carnivores flexibility to use shared areas efficiently, specifically when risk of detection is lowest. The amount of time an animal spends within a habitat also can provide cues as to how and why an animal prefers to spend time within specific sites^21^. For example, large carnivores are generally expected to move straighter and faster within human-dominated landscapes^22,23^ to avoid risk of detection.

Movement parameters can provide detailed knowledge of animal behavior with respect to humans, co-predators and livestock^24^. Both intrinsic (age, sex, life stage) and extrinsic (environmental conditions, availability of resources) factors can contribute to variance in movement^23,25^. Ecological attributes such as availability of water, human presence, and the proximity to protected areas, forests, roads, and crop fields have been reported to affect hunting success and habitat utilization by large carnivores^26–29^. These features are essential for maintaining spatial–temporal avoidance of humans and livestock. In east Africa, lions (*Panthera leo*) demonstrated temporal partitioning with humans and exhibited seasonal and temporal differences in habitat selection based on human activity^12–14^, whereas cheetahs (*Acinonyx jubatus*) were nocturnal, avoided humans, and selected semi-closed habitats^30,31^. Jaguars (*Panthera onca*) inhabiting shared landscapes exhibited preference for areas with ambush cover and killed livestock within closed habitats that were proximal to water^32^. Similarly, leopards (*Panthera pardus*) in Kenya spatiotemporally avoided humans^30^. Diurnal activity and use of specific mangrove habitats by tigers (*Panthera tigris*) were responsible for the large number of attacks on humans in Sundarban delta, India^33^.

Human-wildlife conflicts are severe in India where protected areas are small and interspersed with crop fields, forest reserves, and human settlements. With extensive spatial overlap between large carnivores and humans, and an average density of 380 humans per km^2^ (Census of India 2011, data accessed on May 2020), conserving large carnivores and prevention of human-carnivore conflicts is a formidable challenge in India. In particular, leopards inhabit 68% of the country’s land mass and co-occur with a population of 1.3 billion people^34^. Human-leopard conflicts have increased over the past few decades, leading to frequent attacks on people and livestock, and in many cases retaliatory killings of leopards^35,36^. Considering that leopards are the most adaptive large felid to coexist with humans and livestock^37^ we expect some specific behavioral adjustments to avoid detection. Indeed, the only radio-telemetry study in India documented leopards to be more nocturnal in areas highly populated with humans^17^.

Given the paucity of data on leopard space use patterns within shared landscapes in India, our goal was to quantify how anthropogenic factors influence the activity patterns and spatial ecology of leopards and relative to the occurrence of human-leopard conflicts. Here we use radio-telemetry data to examine leopard habitat use and movement behavior with respect to human activity within a heterogeneous landscape of North Bengal, eastern India. Human-carnivore conflicts are common in this region, with an average of 70 human injuries per year from 2004 to 2016 due to leopard attacks. The majority of these attacks were diurnal in nature, and occurred during the dry season (summer and winter months) within tea plantations^36^. Major victims of leopard attacks were middle-aged tea estate workers who were working in small groups. Livestock depredation events also are common, and occur diurnally during the dry season and within tea plantations^38^. Leopards killed adult cows and goats and livestock killing occurred within tea plantations. Probability of leopard attacks on humans and livestock increased in areas within a heterogeneous matrix of closed and open habitats (i.e. forest, scrubland and tea plantations) and vicinity of protected areas^36,38^. Based on the inferences drawn from these studies, we hypothesize that behavioral adjustments of leopards in response to people and livestock should be influenced by land use types such as tea plantations that provide adequate crop cover and should be the major driver of human-leopard conflicts. We also hypothesize that there would be a spatial shift in space use by leopards between the wet and dry seasons, leading to increased likelihood of conflicts during the dry season. We further hypothesize that leopards should exhibit temporal partitioning of activity based on human activity periods to minimize interactions with humans.

## Results

### Home ranges of radio-collared leopards

**Figure 1:**
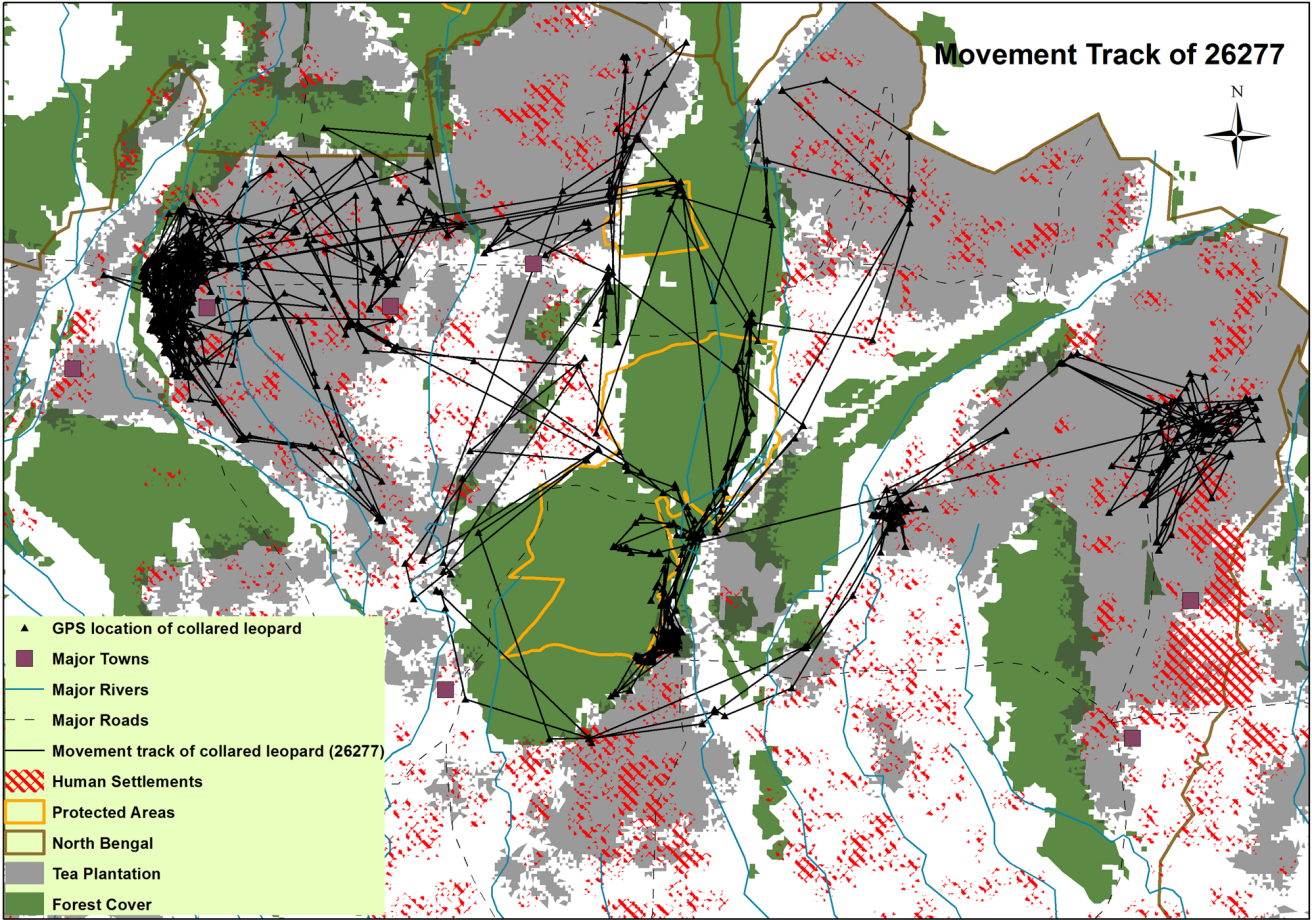
Movement track from Global Positioning System (GPS) data of one sub-adult female leopard (26,277) within different habitat types.

After being collared, two leopards (26,283 and 26,284) exhibited dispersal movement patterns for several weeks before establishing a home range, and all locations collected during these exploratory periods were removed prior to home range estimation, movement, and resource selection analyses. Four leopards were considered for the wet season analyses, 26,279 had no locations for the wet season whereas 32,692 was discarded since it had only 20 locations during this season. For the dry season analyses, five leopards were considered; 32,694 was discarded since it had no locations during this season. Figure 1 shows a visualization of the movement track of sub-adult female leopard 26,277 within different habitat types.

**Figure 2:**
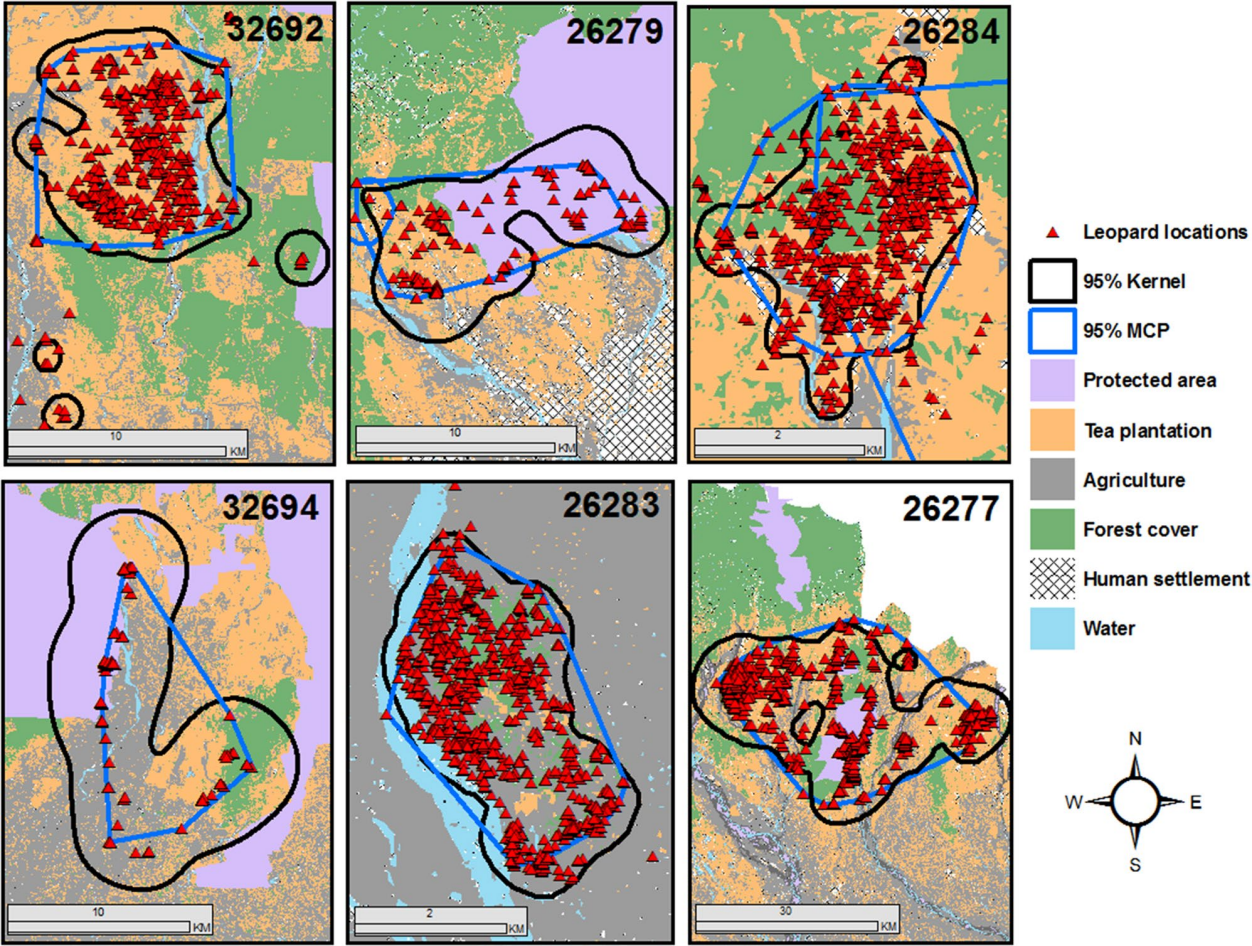
Home ranges of radio-collared leopards in North Bengal prepared using Arc GIS 10.3 (https://enterprise.arcgis.com/en/portal/10.3/use/link-to-items.htm).

A total of 6094 locations from Dec 2017 to April 2020 for 6 leopards were available for the analyses. The average home range size as measured by the 95% MCPs for the collared leopards was 177.47 km^2^ (SD = 324.21) whereas 95% FK home ranges were 212.04 km^2^ (SD = 351.49) (Table 1, Fig. 2). The average core area of leopards (50% FK) was estimated to be 36.66 km^2^ (SD = 53.87). The average home range (95% MCP) during wet season was 104.85 km^2^ (SD = 155.03) whereas 95% FK home ranges were 146.02 km^2^ (SD = 188.16). Average home range (95% MCP) during dry season was 218.51 km^2^ (SD = 358.17) whereas 95% FK home ranges were 275.19 km^2^ (SD = 438.88).

**Table 1:** Individual leopard home ranges from North Bengal, India, including 95% and 50% minimum convex polygon (MCP) estimations, and 95% and 50% fixed kernel (FK) estimations.

Individuals	95% MCP (km^2^)	95% FK (km^2^)	50% MCP (km^2^)	50% FK (km^2^)	Duration	Numbers of locations
26,283 (Sub-adult Male)	7.99	9.09	3.12	2.41	December 2017–January 2018	1490
26,279 (Adult Male)	62.44	87.08	15.85	14.01	January–March 2018	336
26,284 (Adult Female)	6.78	7.08	2.10	1.51	January–August 2018	1326
26,277 (Sub Adult Female)	836.09	919.73	447.44	142.49	August 2019–March 2020	1864
32,694 (Adult Male)	75.28	162.23	45.74	41.80	October 2019–March 2020	151
32,692 (Male)	76.26	87.05	22.89	17.68	August 2019–October 2020	863
Average HR	177.47	212.04	89.52	36.66		1005
SD	324.21	351.49	176.07	53.87		674.26

### Leopard movement and speed within different land use types

Distribution of leopard locations differed significantly across the five habitat types (χ^2^ = 77.2, df = 4, *p* < 0.001). Leopards preferred tea plantations (52%) followed by agriculture fields (24%), forest patches (14%), protected areas (8%), and human settlements (2%). On an average, leopards moved at a speed of 0.229 km/hr (SD = 0.40), although there was no significant variation between habitat types (χ^2^ = 0.94, df = 4, *p* = 0.92). Leopards moved at an average speed of 0.59 km/hr (SD = 1.36) through tea plantations, followed by protected areas 0.20 km/hr (SD = 0.34), forest patches 0.13 km/hr (SD = 0.55), agriculture 0.10 km/hr (SD = 0.21) and human settlements 0.03 km/hr (SD = 0.077). Eighty-percent of the fixes within tea plantations were located within a distance of 100 m, whereas for human habitation, 35% of the fixes were in close proximity (i.e. 200 m of settlements). Around 65% of the fixes were also located within a close proximity (i.e. 200 m) of agriculture fields. Only 19% of the fixes were located within a proximity of 1000 m from protected areas. The average distance moved by leopards per day was estimated to be 0.75 km (SD = 0.71, Range 1.28–0.52). 32,692 adult male travelled the highest average distance per day among the collared leopards.

### Movement behavior

Our dynamic programming algorithm identified two behavioral states exhibited by leopards based on the 4-h time scale data. We identified 2 behavioral states, one stationary behavior ‘resting’ indicative of resting and/or foraging and ‘travelling’ behavior indicative of long, straight movements with high acceleration. However, due to the coarse nature of the data (i.e. moderate temporal scale) we don’t consider the range of behaviors the individual leopards might have engaged in between sampled locations.

Mean step length and turn angle (radians) during stationary ‘resting’ phase/behavior was estimated to be 0.01 km (SD = 0.01) and − 0.07 (SD 2.02) whereas for movement ‘travelling’ behavior it was 1.20 km (SD = 1.53) and − 0.06 (SD = 1.97) (Supplementary Table S1, Supplementary Figure S1, S2, S3).

### Season

**Figure 3:**
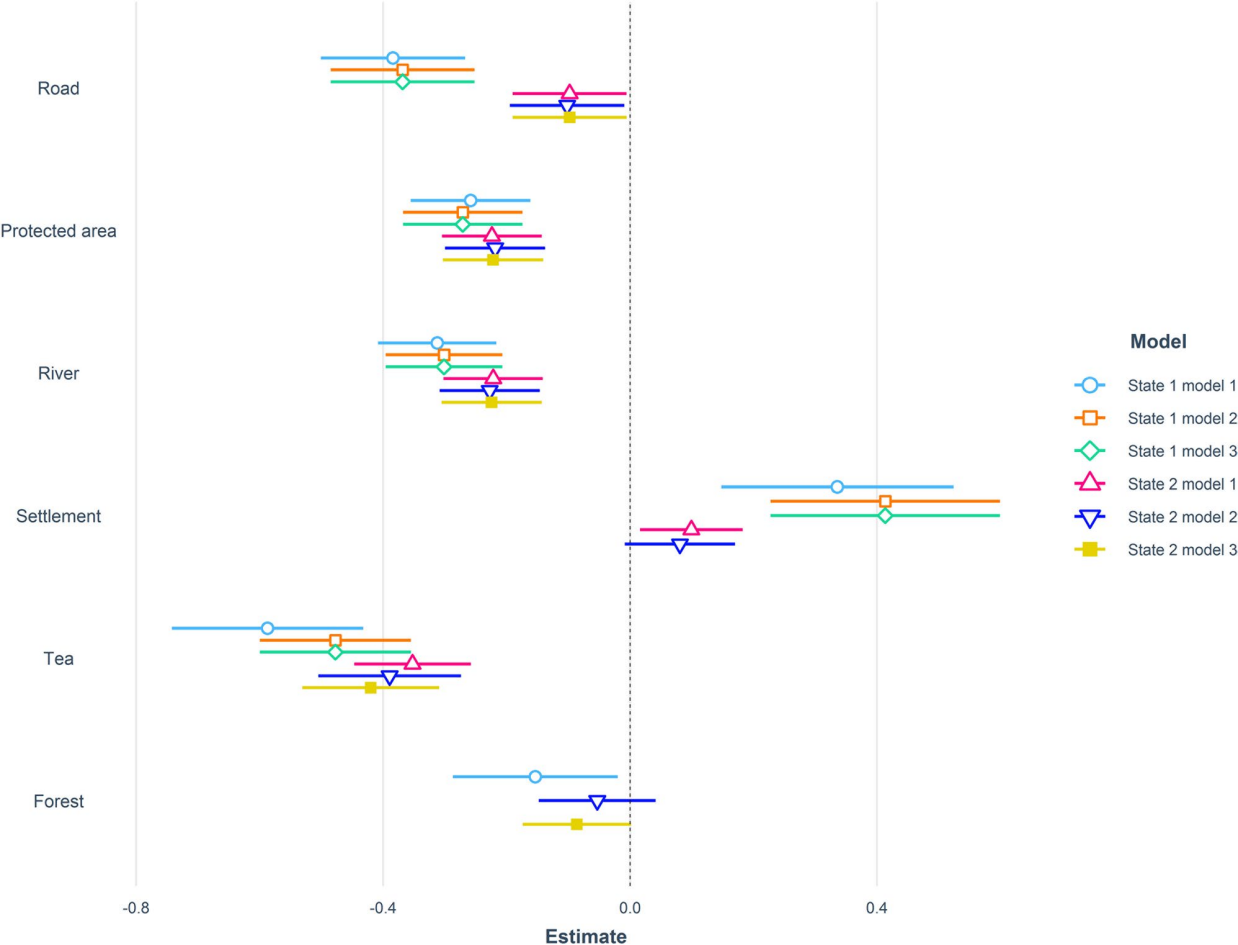

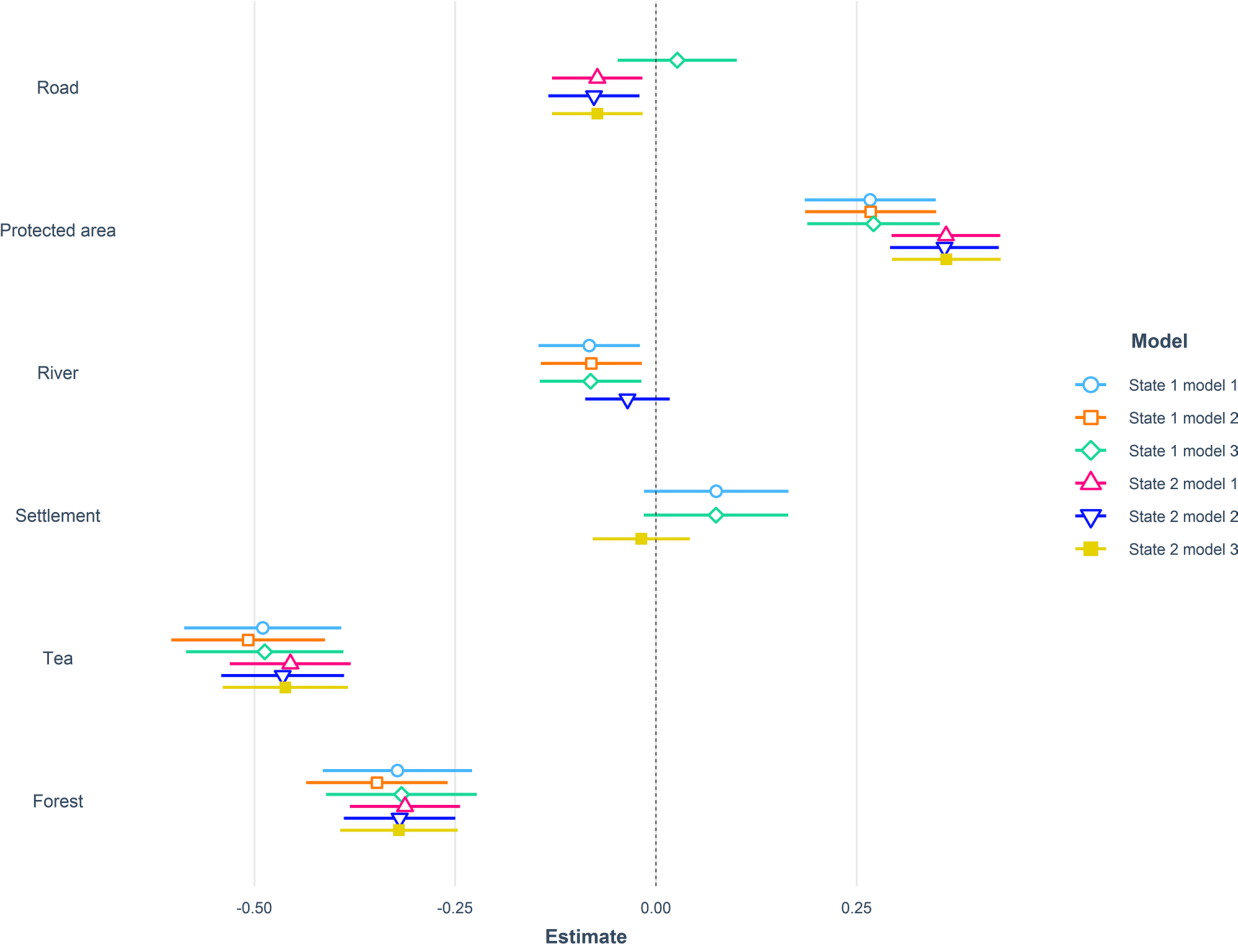
(**A**) Parameter estimates for step-selection function analyses conducted at the 4-h timescale for radio-collared leopards. Results of the three top ranked models conducted on different behavioral subsets within wet season are shown in different colors. (**B**) Parameter estimates for step-selection function analyses conducted at the 4-h timescale for radio-collared leopards. Results of the three top ranked models conducted on different behavioral subsets within dry season are shown in different colors.

During the wet season when human-leopard conflicts are low, leopards showed preference for areas in the vicinity of roads, protected areas, rivers, tea plantations, and forests but avoided human settlements (Supplementary Table S2). The three top ranked models included 99% of the model weight (Table 2, Fig. 3A). Leopards selected sites closer to roads, protected areas, rivers, tea plantations, and forests while resting during the wet season but avoided human settlements (Table 2). The three top ranked models included 100% of the model weight (Table 2). While travelling during the wet season leopards preferred areas near roads, protected areas, rivers, and tea plantations but avoided settlements. The three top ranked models included 72% of the model weight (Table 2).

**Table 2:** Model selection results relating leopard resource selection during the wet (June–October) and dry (November–May) seasons to predictor variables for leopards tracked with GPS collars in North Bengal, India from 2017 to 2020. State 1 represents resting locations whereas state 2 represents travelling locations.

Model	AIC_c_	ΔAIC_c_	*w*_*i*_	LL
**Wet Season**				
Dist. Forest + Dist. PAs + Dist. Road + Dist. River + Dist. Settlement + Dist. Tea plantation	5625.59	0.00	0.88	− 2804.78
Dist. PAs + Dist. Road + Dist. River + Dist. Settlement + Dist. Tea plantation	5630.06	4.46	0.09	− 2808.02
Dist. Forest + Dist. PAs + Dist. Road + Dist. River + Dist. Tea plantation	5632.86	7.26	0.02	− 2809.42
**Dry Season**				
Dist. Forest + Dist. PAs + Dist. Road + Dist. River + Dist. Tea plantation	10,726.02	0.00	0.24	− 5356.01
Dist. Forest + Dist. PAs + Dist. River + Dist. Tea plantation	10,726.29	0.27	0.21	− 5357.14
Dist. Forest + Dist. PAs + Dist. Tea plantation	10,727.04	1.01	0.15	− 5358.51
Dist. Forest + Dist. PAs + Dist. Road + Dist. Tea plantation	10,727.30	1.25	0.14	− 5357.63
Dist. Forest + Dist. PAs + Dist. Road + Dist. River + Dist. Settlement + Dist. Tea plantation	10,728.00	2.00	0.10	− 5356.01
**Wet State 1**				
Dist. Forest + Dist. PAs + Dist. Road + Dist. River + Dist. Settlement + Dist. Tea plantation	3003.88	0.00	0.82	− 1493.92
Dist. PAs + Dist. Road + Dist. River + Dist. Settlement + Dist. Tea plantation	3006.88	3.00	0.18	− 1496.42
Dist. Forest + Dist. PAs + Dist. road + Dist. river + Dist. Tea plantation	3018.36	14.47	0.00	− 1502.16
**Wet State 2**				
Dist. PAs + Dist. Road + Dist. River + Dist. Settlement + Dist. Tea plantation	4561.68	0.00	0.35	− 2273.82
Dist. Forest + Dist. PAs + Dist. Road + Dist. River + Dist. Settlement + Dist. Tea plantation	4562.47	0.79	0.24	− 2273.21
Dist. Forest + Dist. PAs + Dist. Road + Dist. River + Dist. Tea plantation	4563.71	2.03	0.13	− 2274.84
**Dry State 1**				
Dist. Forest + Dist. PAs + Dist. River + Dist. Settlement + Dist. Tea plantation	5771.16	0.00	0.24	− 2878.57
Dist. Forest + Dist. PAs + Dist. River + Dist. Tea plantation	5772.04	0.87	0.17	− 2880.01
Dist. Forest + Dist. PAs + Dist. Road + Dist. River + Dist. Settlement + Dist. Tea plantation	5772.67	1.51	0.11	− 2878.32
**Dry State 2**				
Dist. Forest + Dist. PAs + Dist. Road + Dist. Tea plantation	8689.26	0.00	0.34	− 4338.62
Dist. Forest + Dist. PAs + Dist. Road + Dist. River + Dist. Tea plantation	8689.51	0.26	0.30	− 4337.75
Dist. Forest + Dist. PAs + Dist. Road + Dist. settlement + Dist. Tea plantation	8690.91	1.66	0.15	− 4338.45
Dist. Forest + Dist. PAs + Dist. Road + Dist. River + Dist. settlement + Dist. Tea plantation	8691.20	1.96	0.13	− 4337.60

*Dist.-Distance to.

*PAs- Protected Areas.

During the dry season, leopards selected sites closer to tea plantations and forests but avoided protected areas (Supplementary Table S3). The three top ranked models included 60% of the model weight (Table 2, Fig. 3B). While resting during the dry season leopards preferred areas near rivers, tea plantations, and forests but avoided protected areas. The three top ranked models included 52% of the model weight (Table 2). However, while travelling through the landscape during the dry season, leopards selected areas closer to roads, tea plantations, and forests and avoided human settlements. The three top ranked models included 79% of the model weight (Table 2).

### Human activity

**Figure 4:**
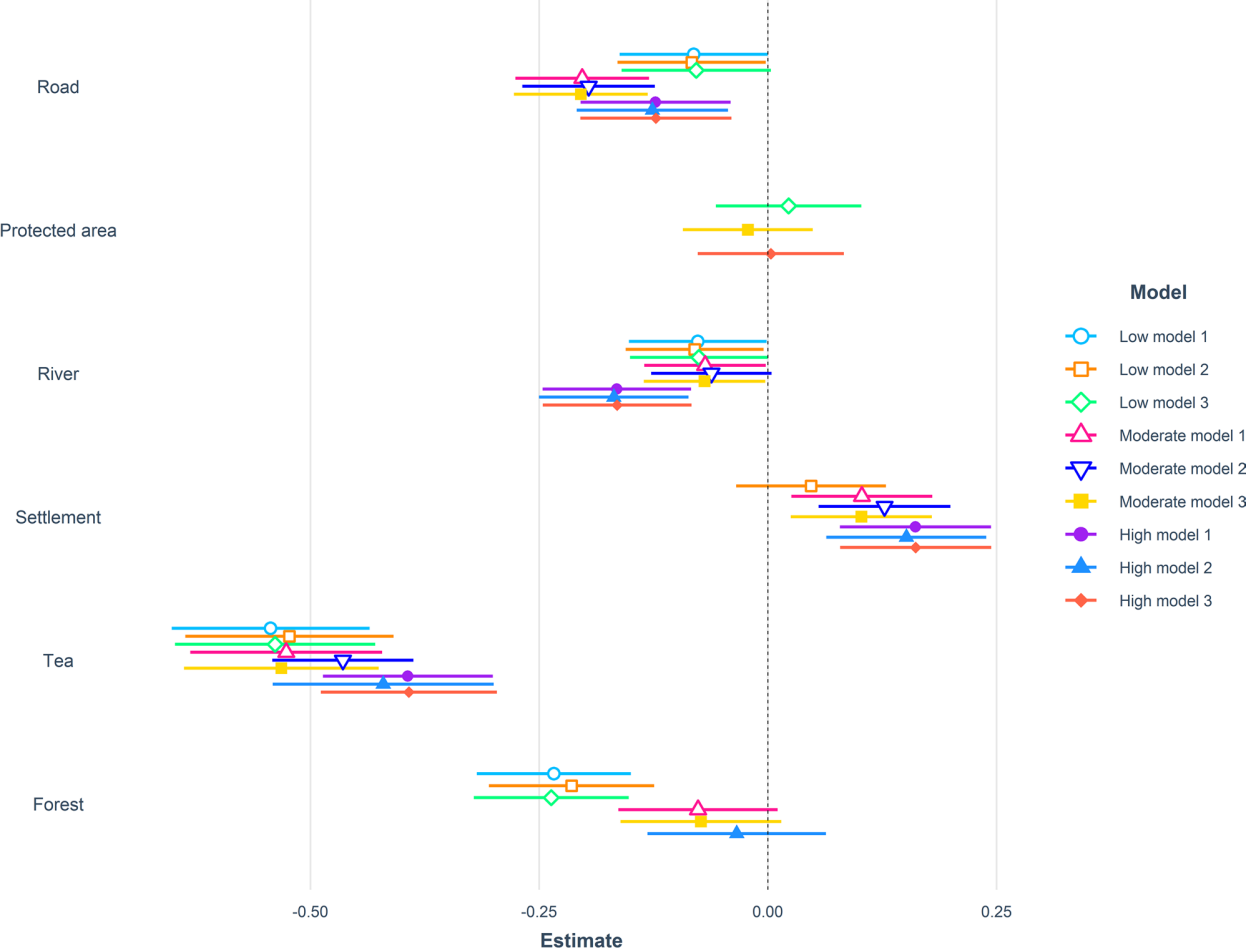
Parameter estimates for step-selection function analyses conducted at the 4-h timescale for radio-collared leopards. Results of the three top ranked models conducted on different behavioral subsets in relation to human activity periods (low, medium, high) are shown in different colors.

During low human activity period (10 PM–6 AM) when risk of encountering humans was low, leopards selected habitats close to roads, rivers, tea plantations, and forests (Supplementary Table S4). The three top-ranked models contained 45% of the model weight (Table 3, Fig. 4). During moderate human activity period (6 AM–10 AM & 6 PM–10 PM), when risk of encountering humans were intermediate leopards preferred habitats close to roads, rivers, and tea plantations but avoided human settlements (Supplementary Table S5). The three top ranked models contained 58% of the model weight (Table 3, Fig. 4). During the high human activity period (10 AM–6 PM), leopards selected habitats close to roads, rivers, and tea plantations but avoided human settlements (Supplementary Table S6). The three top ranked models contained 88% of the model weight (Table 3, Fig. 4). While resting (State 1, (Supplementary Table S7) and travelling (State 2, Supplementary Table S7) during low, moderate and high human activity periods leopards preferred sites close to roads, rivers, forests, tea plantations but avoided human settlements. After accounting for the effect of individual leopards as a random error, we found significant effect of time (estimate: 0.70, SE = 0.02, *p* < 0.001) but not season (estimate: − 0.03, SE = 0.03, *p* = 0.24) on leopard movement.

**Table 3:** Model selection results relating leopard resource selection during periods of low, moderate, and high human activity to predictor variables based on leopards tracked with GPS collars in North Bengal, India from 2017 to 2020. State 1 represents resting locations whereas state 2 represents travelling locations.

Model	AIC_c_	ΔAIC_c_	*w*_*i*_	LL
**Low Human Activity**				
Dist. Forest + Dist. Road + Dist. River + Dist. Tea	5019.539	0	0.210983	− 2503.76
Dist. Forest + Dist. Road + Dist. River + Dist. Settlement + Dist. Tea plantation	5020.246	0.707	0.148	− 2503.11
Dist. Forest + Dist. PAs + Dist. road + Dist. River + Dist. Tea plantations	5021.231	1.692	0.091	− 2503.60
Dist. Forest + Dist. River + Dist. Tea plantations	5021.50	1.93	0.12	− 2505.73
Dist. Forest + Dist. Road + Dist. Tea plantations	5021.50	1.99	0.12	− 2505.76
**Moderate Human Activity**				
Dist. Forest + Dist. Road + Dist. River + Dist. Settlement + Dist. Tea plantation	6018.842	0	0.285	− 3002.41
Dist. Road + Dist. River + Dist. Settlement + Dist. Tea plantation	6019.798	0.956	0.177	− 3003.89
Dist. Forest + Dist. PAs + Dist. Road + Dist. Settlement + Dist. Tea plantation	6020.488	1.645	0.125	− 3002.23
**High Human Activity**				
Dist. Road + Dist. River + Dist. Settlement + Dist. Tea plantation	4682.584	0	0.482	− 2335.28
Dist. Forest + Dist. Road + Dist. River + Dist. settlement + Dist. Tea plantation	4684.121	1.537	0.224	− 2335.04
Dist. PAs + Dist. Road + Dist. River + Dist. Settlement + Dist. Tea plantation	4684.586	2.001	0.177	− 2335.28
**Low State 1**				
Dist. Forest + Dist. Road + Dist. River + Dist. Settlement + Dist. Tea plantation	2168.231	0	0.277	− 1077.09
Dist. Forest + Dist. Road + Dist. River + Dist. Tea plantation	2168.526	0.295	0.239	− 1078.24
Dist. Forest + Dist. PAs + Dist. Road + Dist. River + Dist. Settlement + Dist. Tea plantation	2169.68	1.449	0.135	− 1076.81
Dist. Forest + Dist. PAs + Dist. Road + Dist. River + Dist. Tea plantation	2170.00	1.75	0.13	− 1077.96
**Low State 2**				
Dist. Forest + Dist. Road + Dist. Tea plantation	4367.119	0	0.128	− 2178.55
Dist. Forest + Dist. Tea plantation	4367.322	0.203	0.116	− 2179.65
Dist. Forest + Dist. PAs + Dist. Tea plantation	4367.717	0.597	0.095	− 2178.85
Dist. Forest + Dist. Road + Dist. River + Dist. Tea plantation	4367.80	0.66	0.09	− 2177.87
Dist. Forest + Dist. PAs + Dist. Road + Dist. Tea plantation	4368.00	0.85	0.08	− 2177.97
Dist. Forest + Dist. River + Dist. Tea plantation	4368.40	1.26	0.07	− 2179.18
Dist. Forest + Dist. PAs + Dist. Road + Dist. River	4368.70	1.60	0.06	− 2177.34
Dist. Forest + Dist. PAs + Dist. River + Dist. Tea plantation	4368.80	1.71	0.06	− 2178.40
Dist. Forest + Dist. Road + Dist. Settlement + Dist. Tea plantation	4368.90	1.76	0.05	− 2178.43
**Moderate State 1**				
Dist. Road + Dist. River + Dist. Settlement + Dist. Tea plantation	3566.103	0	0.296	− 1777.04
Dist. Forest + Dist. Road + Dist. River + Dist. Settlement + Dist. Tea plantation	3566.165	0.061	0.287	− 1776.06
Dist. PAs + Dist. Road + Dist. River + Dist. Settlement + Dist. Tea plantation	3567.918	1.814	0.119	− 1776.94
**Moderate State 2**				
Dist. Road + Dist. River + Dist. Settlement + Dist. Tea plantation	4652.433	0	0.163	− 2320.2
Dist. Road + Dist. Settlement + Dist. Tea plantation	4652.604	0.171	0.150	− 2321.29
Dist. Forest + Dist. Road + Dist. River + Dist. Settlement + Dist. Tea plantation	4653.897	1.465	0.078	− 2319.93
Dist. Road + Dist. Tea plantation	4654.00	1.54	0.13	− 2322.98
Dist. PAs + Dist. Road + Dist. River + Dist. Settlement + Dist. Tea plantation	4654.30	1.85	0.11	− 2320.13
Dist. Forest + Dist. Road + Dist. Settlement + Dist. Tea plantation	4654.30	1.87	0.06	− 2321.14
Dist. Forest + Dist. Road + Dist. River + Dist. Tea plantation	4654.30	1.89	0.06	− 2321.15
**High State 1**				
Dist. Road + Dist. River + Dist. Settlement + Dist. Tea plantation	2737.66	0	0.472	− 1362.81
Dist. Forest + Dist. Road + Dist. River + Dist. Settlement + Dist. Tea plantation	2739.228	1.567	0.216	− 1362.59
Dist. PAs + Dist. Road + Dist. River + Dist. Settlement + Dist. Tea plantation	2739.326	1.665	0.205	− 1362.64
**High State 2**				
Dist. Road + Dist. River + Dist. Settlement + Dist. Tea plantation	3803.657	0	0.375	− 1895.81
Dist. Forest + Dist. Road + Dist. River + Dist. Settlement + Dist. Tea plantation	3805.121	1.464	0.181	− 1895.54
Dist. PAs + Dist. Road + Dist. River + Dist. Settlement + Dist. Tea plantation	3805.583	1.925	0.143	− 1895.77

## Discussion

Our study integrated animal locations, remote sensing, and statistical modelling to make inferences regarding the ecological and anthropogenic attributes influencing leopard movement behavior and habitat selection within a human-dominated landscape of South Asia. Our primary focus was to assess how leopards use mixed landscapes during two seasons (dry and wet) and periods of human activity (low, medium, high), and its relevance to human-leopard conflicts in the North Bengal region of India. We identified two major behavioral states (resting and travelling) for the collared leopards. Our results support the first two hypotheses regarding preferential use of anthropogenic habitats and seasonal variation in space use by leopards. We found the majority of leopard locations occurred within close proximity to tea plantations. Resource selection was strongly governed by presence of vegetation cover, proximity to water and avoidance of human settlements. During the dry season when human-leopard conflicts are high^36,37^, leopards actively selected tea plantations and forest patches but avoided protected areas. However, during the wet season, leopards showed preference for forested patches, protected areas, riverine patches, roads, and tea plantations, and avoided settlements. Our 3rd hypothesis was rejected as there was no temporal partitioning between leopards and humans. Leopards showed a preference for sites in close proximity to roads, rivers and tea plantations during periods of moderate and high human activity. Our overall results indicate that there was no spatial–temporal avoidance of humans by leopards, which likely is the major driver of conflicts in the North Bengal landscape. However, there is limited data availability on the spacing and resource selection patterns of leopards that were not radio-tagged in the landscape and hence all results regarding overall habitat use should be interpreted with caution.

The average home range size of collared leopards in our study (177.47 km^2^) was much larger than the previous estimates reported from other Asian landscapes such as India, Nepal^17,39^ and Iran^40^. However, there was also considerable variation in size of home ranges with the lowest being 6.78 km^2^ and the highest being 836 km^2^. The sub-adult leopardess 26,277 had the largest home range as she traversed vast areas between protected areas, tea plantations, crop fields and settlements from the western to the eastern part of the landscape. Home range sizes are an artifact of the spatial distribution and abundance of wild prey^41,42^. The smallest leopard home ranges are reported from the alluvial flood plains of Nepal^39^ and eastern, southern Africa^43,44^ where there is abundance of wild prey, whereas the largest reported home ranges prior to this study (103 km^2^) were reported from Iran^40^.

The interplay between human, animal behavior, activity patterns and resource use has often been regarded as a major driver of human-carnivore conflicts^4^. Within shared landscapes where density of humans is high, some overlap is expected as complete avoidance between humans, livestock and carnivores is not possible. Leopards showed flexibility in using anthropogenic areas and preferred a matrix of both open and closed habitats. Carnivores are expected to move faster and straighter within anthropogenic landscapes^13,45^. Our collared leopards exhibited no significant variation in speed while travelling across habitat types such as tea plantations, crop fields, settlements, forests and protected areas. Though human settlement areas provide easy prey such as unattended livestock, domestic dogs^46,47^ there was no major variation in movement within this habitat. There was similarity in the relationship between the seasonal use of sites with close proximity to tea plantations, rivers, and avoidance of settlements across both the behavioral states. While moving and resting, leopards preferred habitats with vegetation cover and water, but avoided areas close to human settlements. Such results indicate there was an avoidance of sites with absence of crop cover such as settlements, which seems to be an artifact of their perception of risk (landscape of fear)^48^. Though leopards are the most adaptive large felid^37^, they also exhibit fear of human presence and hence avoid settlements. Our findings are similar to a global study on movement behavior of a diversity of large mammals^49^. Cheetahs and lions also avoided areas with increased human disturbance in Kenya^14,50^, which suggests human presence and disturbance can have a negative influence on the movement behavior of large carnivores.

Our data were limited to a four-hour collection interval, and thus we were unable to distinguish between resting and feeding behaviors within our stationary state, although given the foraging behavior of leopards the majority of stationary locations likely reflected resting behavior.

Large carnivores generally prefer areas with dense to moderate vegetation cover to avoid threats from co-predators and humans^14,31,33,51^. Though leopards are considered to be habitat generalists^45^_,_ they are reported to exhibit preference for landscape features which provide concealment within human-dominated landscapes^52,53^. Tea plantations and riverine habitats are interspersed between protected areas, crop fields and forest patches and provide cover within a human-dominated landscape.

Our earlier study findings highlight that leopards attacked humans and livestock in North Bengal primarily during the dry season^36,37^. During the dry season, tea laborers work intensively within the gardens and engage in various activities which includes plucking of leaves, pesticide control, sprinkling of water, removal of old growth/mature stands and adding fertilizer to the plants^36^. Livestock density was fairly high with an average density of 340 animals/km^2^ and leopard diet was also reported to be primarily comprised of livestock in this region^47^. Data on wild prey availability within the landscape was extremely limited in nature and hence the results concerning resource selection by leopards should be interpreted with caution. Assessing seasonal abundance of wild prey, livestock, dogs, and rodents within tea plantations, riverine and forest patches will be an important topic for future research efforts in this landscape.

Our generalized linear mixed model results suggested there was significant variation in leopard movement behavior between night and day. Leopards utilized high risk (conflict prone) areas such as tea plantations during all time periods but avoided settlements especially during moderate and high human activity (8 AM–4 PM). Leopard attacks on humans and livestock often occur within or near tea plantations in our study area, typically during the day when workers are present within the gardens with peak attacks between (8 AM–4 PM). Attacks also are higher in areas with a matrix of open habitats and vegetation cover^36,37^, suggesting humans may be incidentally encountering resting leopards when working in tea plantations. Carnivores optimize their behavior based on the risk of mortality from other predators and humans^54,55^. In Tanzania where risk of mortality was low, lions were attracted to multiple-use areas outside protected reserves due to presence of wild pigs (*Sus scrofa*), resulting in attacks on humans at night^56^. However, in Kenya where risk of persecution by humans was high, lions avoided areas close to settlements, water, and pastoral lands, and used dense vegetation cover during high human activity periods^13,14,57^. In the Sundarban delta where risk of persecution was negligible, tiger attacks on humans were documented to be seasonal and diurnal, and coincided with the activity patterns of honey collectors and fishermen^33^. In central India and Terai region of Nepal, there was no seasonal variation in leopard attacks on humans and the majority occurred near forest edges at the interface of human and carnivore activity^58,59^. Similarly, in Thailand and Nepal, leopards avoided areas of human disturbance such as roads during the day^60,61^. Within a multiple-used landscape of Northern Kenya, leopards avoided areas close to roads and human presence during the day and used vegetation cover for concealment^53^. The only radio-telemetry study from India documented that leopards avoided areas of high human presence during the day but were close to settlements during night in search of domestic dogs and unsupervised livestock^17^.

We acknowledge some limitations of our study such as the low sample size and the coarse 4-h duration of fixes. However, this is the first detailed radio-telemetry study on the movement behavior and space use by leopards in South Asia, and thus provides unique insights into the behavioral strategies employed by leopards to access resources within a heterogeneous shared landscape of eastern India and its implications for mitigation of human-leopard conflicts. Our results demonstrate that there were spatial–temporal overlaps in activity and resource selection by leopards and humans. Leopards didn’t exhibit complete avoidance of anthropogenic areas and preferred tea plantations. Due to a strong cultural reverence towards wildlife, there was low risk of persecution by humans in this landscape. Unlike other large carnivores, leopards showed behavioral adjustments and selected for areas with vegetation cover within the fragmented, multiple-use North Bengal landscape. A combination of reduced movement and concealment during high to moderate human activity periods allowed leopards to remain undetected. However, such behavioral and landscape related preference in space use and activity could be the major driver of human-leopard conflicts in this region. With increasing incidents of attacks on tea estate workers and predation on livestock every year, the costs of co-existence with leopards are high. Prolonged costs of damage would reduce cultural tolerance leading to retaliation against leopards.

Hence necessary measures such as development of early warning systems through digital-geo fencing of conflict prone areas, real time GPS tracking of leopards and livestock, modifying behavior of tea estate workers through conservation outreach programs, use of non-lethal carnivore deterrents and recognizing costs of coexistence with large carnivores should be initiated within conflict hotspots. Animal husbandry practices such as supervised grazing of livestock should be adopted, use of protective collars and knowledge of habitats preferred by leopards should be discussed and community members should be engaged as leopard custodians to monitor carnivore presence around settlements. Further research should be undertaken to understand fine scale behavioral response of leopards within anthropogenic habitats and protected areas, the impact of appropriate herding practices, and the efficacy of conservation outreach programs on reducing livestock losses and attacks on humans. Finally, recognizing the importance of shared landscapes in sustaining large carnivore populations is crucial to ensure coexistence as human expansion and activities continue to modify habitats within developing regions of South Asia.

## Methods

### Study area

**Figure 5:**
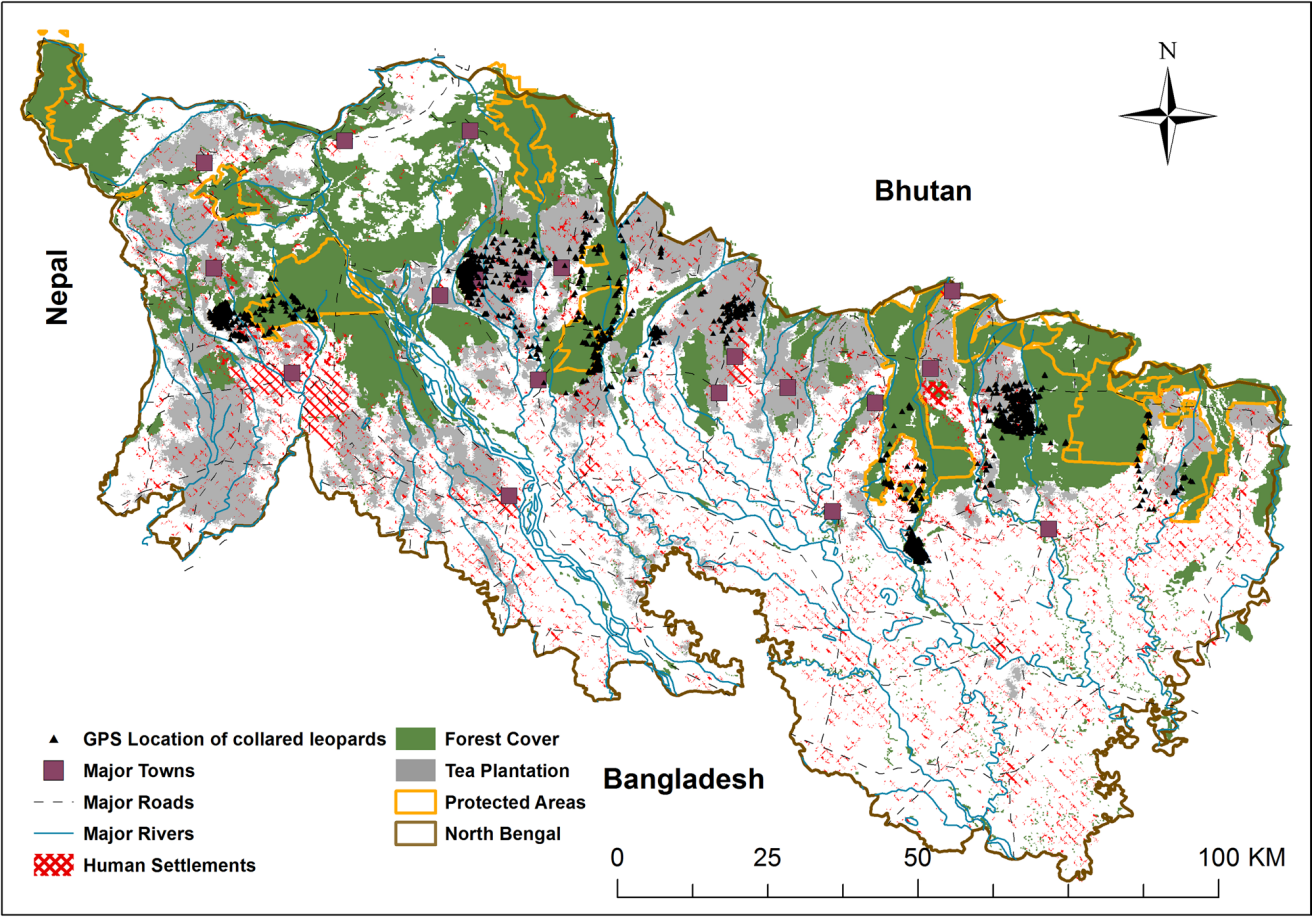
Location of North Bengal within India along with gps fixes of collared leopards, protected areas, major roads, towns, rivers, human settlements, tea plantations and forest cover prepared using Arc GIS 10.3 (https://enterprise.arcgis.com/en/portal/10.3/use/link-to-items.htm).

The study was conducted within the human-dominated North Bengal landscape in eastern India (Fig. 5). The study area (12,500 km^2^) included both wildlife and non-wildlife areas. The areas allocated for wildlife included protected areas such as Buxa National Park (761 km^2^), Jaldapara National Park (217 km^2^), Gorumara National Park (80 km^2^), Chapramari Wildlife Sanctuary (9.5 km^2^), and Mahananda Wildlife Sanctuary (158 km^2^)^62^, as well as reserve forests. The human-use areas included tea plantations, agriculture fields, and human settlements. The protected areas were not fenced and hence wild animals could roam freely between protected and non-wildlife areas. The landscape matrix of this region was comprised of forests, agricultural fields, tea plantations, river beds, and human settlements interspersed with protected areas^63^. The region was comprised of 46% forest cover, with moist tropical or sub-tropical forests comprising the predominant forest types along the foothills of the Eastern Himalayas. This region is part of the Terai-Duars belt, which historically was a contiguous forest patch extending from Nepal in the west to Bhutan and Assam hills in the east.

The average human density ranges between 200 and 700 individuals per km^2^ (Census of India 2011, Accessed May 2020). Tribal communities such as Oraon, Munda, Santhals, Bhutia, Rajbanshi and other ethnic groups such as Gorkha, Bengali inhabit this region. Local community members possess livestock and stay within settlements close to forested regions and protected areas. The average livestock density is 340 animals per km^2^. Primary occupations are in agriculture, livestock rearing, daily labors, and tea estates. Tribal community members are engaged as tea estate workers. The tea estates are government lands leased to private companies. These private companies manage the plantations, provide employment to the local communities, and share a small portion of the revenue with the government. The village lands are managed by the local community heads (gram panchayat), whereas settlements close to the protected areas are managed by the forest and wildlife staff. This region is characterized by an annual rainfall range of 1200–3200 mm with 3 distinct seasons i.e. summer (March–June), monsoon (July–October), and winter (November–February). Summer and winter are dry, with the maximum rainfall received during the monsoon season. Thus, we considered two major seasons i.e. dry (summer and winter) and wet (monsoon) for our analyses.

### Collaring of leopards

Six leopards (1adult female, 3 adult males, 1 sub adult male, and 1 sub adult female) were radio-collared between 2017 and 2019. The leopards were trapped in cages using bait and anesthetized using ketamine i.e. 3 mg/kg and xylazine 2 mg/kg administered intra-muscularly using a blow gun^64^. We used live goats to lure leopards towards the cages and all leopard trapping and captures occurred in human-dominated landscapes such as villages and tea plantations within close proximity to protected areas. Animal capture was conducted in collaboration with the Wildlife Wing, Govt. of West Bengal who provided necessary permission and logistic support. All procedures for radio collaring were performed by an experienced veterinarian in collaboration with the wildlife officials. Ethical clearance was sough and studies were performed in accordance with the relevant guidelines and regulations, reviewed and approved by the Wildlife Wing, Govt. of West Bengal (Permit No. 1251/WL/2 W-679/2017 dated 09 March 2017). The leopards were aged based on tooth eruption and wear patterns, development of secondary sexual characters, and genital organs. All leopards were fitted with VERTEX PLUS IRIDIUM GPS collars provided by Vectronic Aerospace (Berlin, Germany). The gps collars weighed less than 1–2% of the body weight of leopards irrespective of the sex. Collars were programmed to record fixes every 4 h and were equipped with a vhf beacon which facilitated ground tracking of the animals (1–13 months). The study was carried out in compliance with the ARRIVE Guidelines.

### Data analysis

#### Home ranges

We cleaned GPS data to exclude spurious locations by mapping the GPS data within ArcMap 10.7.1 (ESRI, 2019) and removing points outside of the study area. We estimated leopard home ranges using Fixed kernel density estimations (KDE) where we extracted 95% and 50% utilization distributions using the ‘adehabitatHR’ package in program R^65^, using a reference bandwidth reduced to a fixed proportion of 0.80 in an effort to reduce over-smoothing^66^. In addition, we quantified 95% minimum convex polygon (MCP) home ranges (calculating overall, wet, and dry season home ranges for each individual) for use in resource selection modelling^67^ (Table 1).

#### Movement behavior

To assess the relationship between leopard movement patterns and anthropogenic land use features, we extracted step lengths, turn angles, and movement states for each leopard location using the ‘moveHMM’ package in program R^68,69^. We calculated the average distance by adding all hourly displacements per day for all individual leopards. Speed of leopard movement was estimated as the distance between consecutive fixes divided by time (km/hr). We tested for a variation in speed among the leopards monitored in response to different situations of risk by comparing speed values among different land-use categories. We compared the results of 25 Hidden Markov Models (HMM), each with randomly generated starting parameters of step lengths and turn angles for each behavioral state, to designate starting parameters that would maximize the log-likelihood of each behavioral state and to assess numerical stability of the models^68^. Because our data were restricted to a four-hour interval between leopard locations, we developed a single HMM incorporating two behavioral states (i.e. stationary and travelling) as it was impractical to tease out additional behavioral states given the resolution of our data. We extracted movement states using the Viterbi algorithm to generate the most likely state associated with each observation. After extracting movement states, we subdivided the data based on seasonal and human activity periods of interest: (1) dry season (November–May), (2) wet season (June–October), (3) low human activity period (10 PM–6 AM), (4) moderate human activity period (6 AM–10 AM and 6 PM–10 PM), and (5) high human activity period (10 AM–6 PM). To examine differences in movement patterns, we further divided each of the above subsections of data by movement state, for a total of 10 subsections of data. We also calculated average movement rates for individual leopards based on distances between consecutive locations, as well as average movement rates within each main habitat type across all individuals. Day and Night were defined as even hour periods (6 AM–6 PM, day) and (6 PM–6 AM, night). Movement of leopards could vary between seasons and time of the day. We analyzed distance moved by individual leopards using a generalized linear mixed model where time was considered as day and night while effect of season was considered as dry and wet. Variation due to individual leopards were considered as a random error in the model. The analysis was done in R using the function ‘glmer’ within the package ‘lme4′ in R^70^.

#### Resource selection

We calculated the proportion of leopard locations in relation to the proximity of gps fixes from different habitats using Arc Map. To investigate whether leopards avoided anthropogenic areas compared to forested and protected habitats, we constructed resource selection functions (RSF) at the home range scale (third order)^71^ by comparing habitat use (estimated from the observed leopard location data) to habitat availability (estimated through randomly generated locations within leopard home ranges). Habitat use analysis incorporated availability and usage of each habitat type by collared leopards. We conducted separate RSF analyses based on the subsections of data outlined previously: (1) dry season, (2) wet season, (3) low human activity periods, (4) moderate human activity periods, and (5) high human activity periods. We generated random points within each leopard’s MCP home range using the ‘spsample’ function in the ‘sp’ package in program R^72^. Each leopard’s overall home range was used as the boundary to generate random points for each of the human activity RSF subsets, whereas the dry and wet season home ranges of each leopard were used as the boundary for those respective subsets. The number of random points generated was equal to the number of observed leopard locations within each subsection of data, resulting in an equal number of used and available locations for each RSF. To examine associations between leopard habitat use and movement behavior, each of the above subsections of data was further divided by movement state for each RSF.

For each used and random location, we quantified the distance to tea plantations, forests, agriculture and human settlements using the land use type map of North Bengal^63^. Since collared leopards occupied flatlands we didn’t consider altitude, slope or terrain ruggedness as a predictor variable for the resource selection analysis. There was also limited data availability on terrain ruggedness for the landscape. We calculated distance to protected areas using the Protected Area Network of India. Distance to rivers and roads were calculated using the Roads and Drainage layers obtained from Digital Chart of the World. We extracted distance covariates using the ‘near’ function within ArcMap 10.7.1^73^. All these variables were log transformed and standardized using z score values with a mean of 0 and a standard deviation of 1. Variables were tested for correlations using Pearson correlation coefficients and the ‘distance to agriculture’ variable was removed because it was correlated (0.67) with ‘distance to human settlement’^74^ (Supplementary Table S8).

We generated RSFs by constructing generalized linear mixed-effect models with individual leopards as a random effect using the ‘glmer’ function within the ‘lme4′ package in program R^70^. We developed a global model incorporating all habitat variables as well as all possible combinations of variables, and selected the best model based on lowest Akaike information criteria corrected for small sample sizes (AIC_c_)^75^.

## Supplementary Information

Supplementary Information.
