# Supplementary material for: Movement behavior of a solitary large carnivore within a hotspot of human-wildlife conflicts in India
Source: Sci Rep. 2021 Feb 16;11:3862. doi: 10.1038/s41598-021-83262-5 (PMC7887241; doi:10.1038/s41598-021-83262-5)
Supplement: Supplementary file 1 — Supplementary Information. [file 41598_2021_83262_MOESM1_ESM.docx]

**Movement behavior and resource selection by a solitary large carnivore within a hotspot of human-wildlife conflicts in North Bengal, India**

**Dipanjan Naha^1^, Suraj Kumar Dash^1^, Caitlin Kupferman^2^, James C Beasley^2^, Sambandam Sathyakumar^1*^**

**Supplementary Table S1 Movement parameters (step lengths, turn angles) of collared leopards**

| **Movement Variable** | **Step Length (km)** | **SD** | **Turn Angle (Radians)** | **SD** |
| --- | --- | --- | --- | --- |
| **Resting behavior (Overall)** | 0.01 | 0.01 | -0.07 | 2.02 |
| **Travelling behavior (Overall)** | 1.20 | 1.53 | -0.06 | 1.97 |
| **Resting behavior (Dry season)** | 0.01 | 0.01 | -0.07 | 2.01 |
| **Travelling behavior (Dry season)** | 1.34 | 1.72 | -0.06 | 1.94 |
| **Resting behavior (Wet season)** | 0.01 | 0.01 | -0.07 | 2.04 |
| **Travelling behavior (Wet season)** | 0.93 | 1.06 | -0.05 | 2.02 |
| **Resting behavior (Low human activity period)** | 0.01 | 0.01 | 0.06 | 2.01 |
| **Travelling behavior (Low human activity period)** | 1.35 | 1.60 | -0.03 | 2.11 |
| **Resting behavior (Moderate human activity period)** | 0.01 | 0.01 | -0.10 | 1.10 |
| **Travelling behavior (Moderate human activity period)** | 1.41 | 1.84 | -0.07 | 1.94 |
| **Resting behavior (High human activity period)** | 0.01 | 0.01 | -0.12 | 2.06 |
| **Travelling behavior (High human activity period)** | 0.74 | 0.76 | -0.074 | 1.82 |

**Supplementary Table S2 Covariate estimates, standard errors (SE), and *P*-values for the top three models of leopard resource selection during the wet season**

| **Wet season** | **Top model** |  |  | **Model 2** |  |  | **Model 3** |  |  |
| --- | --- | --- | --- | --- | --- | --- | --- | --- | --- |
| **Covariates** | **Estimates** | **SE** | **P value** | **Estimates** | **SE** | **P value** | **Estimates** | **SE** | **P value** |
| (Intercept) | 0 | 0.17 | 0.98 | 0 | 0.15 | 0.992 | 0.01 | 0.2 | 0.956 |
| ***Dist. Road** | -0.23 | 0.04 | **<0.001** | -0.22 | 0.04 | **<0.001** | -0.22 | 0.04 | **<0.001** |
| **Dist. *PAs** | -0.24 | 0.04 | **<0.001** | -0.25 | 0.04 | **<0.001** | -0.25 | 0.04 | **<0.001** |
| **Dist. River** | -0.28 | 0.04 | **<0.001** | -0.27 | 0.04 | **<0.001** | -0.28 | 0.04 | **<0.001** |
| **Dist. Settlement** | 0.12 | 0.04 | **0.003** | 0.16 | 0.04 | **<0.001** |  |  |  |
| **Dist. Tea plantation** | -0.48 | 0.06 | **<0.001** | -0.4 | 0.04 | **<0.001** | -0.53 | 0.05 | **<0.001** |
| **Dist. Forest** | -0.11 | 0.04 | **0.011** |  |  |  | -0.16 | 0.04 | **<0.001** |
| **Random Effects** | | | | | | | | | |
| σ^2^ | 3.29 |  |  | 3.29 |  |  | 3.29 |  |  |
| τ_00_ | 0.12 _ID_ |  |  | 0.09 _ID_ |  |  | 0.15 _ID_ |  |  |
| ICC | 0.03 |  |  | 0.03 |  |  | 0.04 |  |  |
| N | 4 _ID_ |  |  | 4 _ID_ |  |  | 4 _ID_ |  |  |
| Observations | 4190 |  |  | 4190 |  |  | 4190 |  |  |
| Marginal R^2^ / Conditional R^2^ | 0.097 / 0.128 |  |  | 0.088 / 0.111 |  |  | 0.103 / 0.141 |  |  |

* Dist.- Distance to

*PAs- Protected Areas

**Supplementary Table S3. Covariate estimates, standard errors (SE), and *P*-values for the top three models of leopard resource selection during the dry season**

| **Dry season** | **Top model** | | | **Model 2** | | | **Model 3** | | |
| --- | --- | --- | --- | --- | --- | --- | --- | --- | --- |
| **Covariates** | **Estimates** | **SE** | **P-value** | **Estimates** | **SE** | **P-value** | **Estimates** | **SE** | **P-value** |
| (Intercept) | 0.02 | 0.15 | 0.889 | 0.02 | 0.14 | 0.877 | 0.02 | 0.14 | 0.871 |
| Dist. Road | -0.04 | 0.03 | 0.133 |  |  |  |  |  |  |
| **Dist. PAs** | 0.31 | 0.03 | **<0.001** | 0.31 | 0.03 | **<0.001** | 0.32 | 0.03 | **<0.001** |
| Dist. River | -0.04 | 0.02 | 0.072 | -0.04 | 0.02 | 0.098 |  |  |  |
| **Dist. Tea plantation** | -0.5 | 0.03 | **<0.001** | -0.5 | 0.03 | **<0.001** | -0.49 | 0.03 | **<0.001** |
| **Dist. Forest** | -0.33 | 0.03 | **<0.001** | -0.32 | 0.03 | **<0.001** | -0.32 | 0.03 | **<0.001** |
| **Random Effects** | | | | | | | | | |
| σ^2^ | 3.29 | | | 3.29 | | | 3.29 | | |
| τ_00_ | 0.10 _ID_ | | | 0.10 _ID_ | | | 0.10 _ID_ | | |
| ICC | 0.03 | | | 0.03 | | | 0.03 | | |
| N | 5 _ID_ | | | 5 _ID_ | | | 5 _ID_ | | |
| Observations | 7958 | | | 7958 | | | 7958 | | |
| Marginal R^2^ / Conditional R^2^ | 0.080 / 0.108 | | | 0.077 / 0.104 | | | 0.077 / 0.104 | | |

**Supplementary Table S4 Covariate estimates, standard errors (SE), and *P*-values for the top three models of leopard resource selection during periods of low human activity**

| **Low human activity** | **Top Model** | | | **Model 2** | | | **Model 3** | | |
| --- | --- | --- | --- | --- | --- | --- | --- | --- | --- |
| **Covariates** | **Estimate** | **SE** | **P value** | **Estimate** | **SE** | **P value** | **Estimate** | **SE** | **P value** |
| (Intercept) | -0.03 | 0.12 | 0.81 | -0.03 | 0.11 | 0.798 | -0.02 | 0.12 | 0.845 |
| Dist. Road | -0.08 | 0.04 | 0.049 | -0.08 | 0.04 | 0.044 | -0.08 | 0.04 | 0.06 |
| **Dist. River** | -0.08 | 0.04 | 0.046 | -0.08 | 0.04 | 0.038 | -0.08 | 0.04 | 0.05 |
| **Dist. Tea plantation** | -0.54 | 0.06 | <0.001 | -0.52 | 0.06 | <0.001 | -0.54 | 0.06 | <0.001 |
| **Dist. Forest** | -0.23 | 0.04 | <0.001 | -0.21 | 0.05 | <0.001 | -0.24 | 0.04 | <0.001 |
| Dist. Settlement |  |  |  | 0.05 | 0.04 | 0.258 |  |  |  |
| Dist. PAs |  |  |  |  |  |  | 0.02 | 0.04 | 0.574 |
| **Random Effects** | | | | | | | | | |
| σ^2^ | 3.29 |  |  | 3.29 |  |  |  |  |  |
| τ_00_ | 0.08 _ID_ |  |  | 0.07 _ID_ |  |  |  |  |  |
| ICC | 0.02 |  |  | 0.02 |  |  |  |  |  |
| N | 6 _ID_ |  |  | 6 _ID_ |  |  |  |  |  |
| Observations | 3686 |  |  | 3686 |  |  |  |  |  |
| Marginal R^2^ / Conditional R^2^ | 0.065/0.086 |  |  | 0.062/0.081 |  |  |  |  |  |

**Supplementary Table S5 Covariate estimates, standard errors (SE), and *P*-values for the top three models of leopard resource selection during periods of moderate human activity**

| **Moderate human activity** | **Top model** | | | **Model 2** | | | **Model 3** | | |
| --- | --- | --- | --- | --- | --- | --- | --- | --- | --- |
| **Covariates** | **Estimate** | **SE** | **P-value** | **Estimate** | **SE** | **P-value** | **Estimate** | **SE** | **P-value** |
| (Intercept) | 0.03 | 0.14 | 0.817 | 0.03 | 0.13 | 0.819 | 0.03 | 0.14 | 0.841 |
| **Dist. Road** | -0.2 | 0.04 | **<0.001** | -0.2 | 0.04 | **<0.001** | -0.2 | 0.04 | **<0.001** |
| **Dist. River** | -0.07 | 0.03 | **0.043** | -0.06 | 0.03 | 0.066 | -0.07 | 0.03 | **0.042** |
| **Dist. Settlement** | 0.1 | 0.04 | **0.009** | 0.13 | 0.04 | **0.001** | 0.1 | 0.04 | **0.009** |
| **Dist. Tea plantation** | -0.53 | 0.05 | **<0.001** | -0.46 | 0.04 | **<0.001** | -0.53 | 0.05 | **<0.001** |
| Dist. Forest | -0.08 | 0.04 | 0.086 |  |  |  | -0.07 | 0.04 | 0.103 |
| Dist. PAs |  |  |  |  |  |  | -0.02 | 0.04 | 0.548 |
| **Random Effects** | | | | | | | | | |
| σ^2^ | 3.29 |  |  | 3.29 |  |  | 3.29 |  |  |
| τ_00_ | 0.10 _ID_ |  |  | 0.08 _ID_ |  |  | 0.11 _ID_ |  |  |
| ICC | 0.03 |  |  | 0.02 |  |  | 0.03 |  |  |
| N | 6 _ID_ |  |  | 6 _ID_ |  |  | 6 _ID_ |  |  |
| Observations | 4482 |  |  | 4482 |  |  | 4482 |  |  |
| Marginal R^2^ / Conditional R^2^ | 0.090 / 0.117 |  |  | 0.083 / 0.106 |  |  | 0.091 / 0.119 |  |  |

**Supplementary Table S6 Covariate estimates, standard errors (SE), and *P*-values for the top three models of leopard resource selection during periods of high human activity**

| **High human activity** | **Top model** | | | **Model 2** | | | **Model 3** | | |
| --- | --- | --- | --- | --- | --- | --- | --- | --- | --- |
| **Covariates** | **Estimate** | **SE** | **P-value** | **Estimate** | **SE** | **P-value** | **Estimate** | **SE** | **P-value** |
| (Intercept) | 0 | 0.09 | 0.971 | 0 | 0.09 | 0.98 | 0 | 0.09 | 0.976 |
| **Dist. Road** | -0.12 | 0.04 | **0.003** | -0.13 | 0.04 | **0.003** | -0.12 | 0.04 | **0.004** |
| **Dist. River** | -0.16 | 0.04 | **<0.001** | -0.17 | 0.04 | **<0.001** | -0.16 | 0.04 | **<0.001** |
| **Dist. Settlement** | 0.16 | 0.04 | **<0.001** | 0.15 | 0.04 | **0.001** | 0.16 | 0.04 | **<0.001** |
| **Dist. Tea plantation** | -0.39 | 0.05 | **<0.001** | -0.42 | 0.06 | **<0.001** | -0.39 | 0.05 | **<0.001** |
| Dist. Forest |  |  |  | -0.03 | 0.05 | 0.494 |  |  |  |
| Dist. PAs |  |  |  |  |  |  | 0 | 0.04 | 0.936 |
| **Random Effects** | | | | | | | | | |
| σ^2^ | 3.29 |  |  | 3.29 |  |  | 3.29 |  |  |
| τ_00_ | 0.04 _ID_ |  |  | 0.04 _ID_ |  |  | 0.04 _ID_ |  |  |
| ICC | 0.01 |  |  | 0.01 |  |  | 0.01 |  |  |
| N | 6 _ID_ |  |  | 6 _ID_ |  |  | 6 _ID_ |  |  |
| Observations | 3452 |  |  | 3452 |  |  | 3452 |  |  |
| Marginal R^2^ / Conditional R^2^ | 0.057 / 0.067 |  |  | 0.058 / 0.070 |  |  | 0.057 / 0.067 |  |  |

| **Low human activity State 1 (Resting)**  **Supplementary Table S7 Covariate estimates, standard errors (SE), and *P*-values for the top three models of leopard resource selection when resting (state 1) and travelling (state 2) during periods of low, moderate and high human activity** | **Top model** | | | **Model 2** | | | **Model 3** | | |
| --- | --- | --- | --- | --- | --- | --- | --- | --- | --- |
| **Covariates** | **Estimate** | **SE** | **P-value** | **Estimate** | **SE** | **P-value** | **Estimate** | **SE** | **P-value** |
| (Intercept) | -1.43 | 0.18 | <0.001 | -1.43 | 0.17 | <0.001 | -1.44 | 0.17 | <0.001 |
| **Dist. Road** | **-0.13** | **0.06** | **0.021** | **-0.13** | **0.06** | **0.028** | **-0.14** | **0.06** | **0.018** |
| **Dist. River** | **-0.16** | **0.06** | **0.005** | **-0.15** | **0.06** | **0.007** | **-0.17** | **0.06** | **0.004** |
| Dist. Settlement | 0.11 | 0.08 | 0.148 |  |  |  | 0.11 | 0.08 | 0.148 |
| **Dist. Tea plantation** | **-0.55** | **0.09** | **<0.001** | **-0.58** | **0.09** | **<0.001** | **-0.55** | **0.09** | **<0.001** |
| **Dist. Forest** | **-0.34** | **0.07** | **<0.001** | **-0.38** | **0.07** | **<0.001** | **-0.33** | **0.08** | **<0.001** |
| Dist. PA |  |  |  |  |  |  | -0.05 | 0.06 | 0.448 |
| **Random Effects** | | | | | | | | | |
| σ^2^ | 3.29 |  |  | 3.29 |  |  | 3.29 |  |  |
| τ_00_ | 0.16 _ID_ |  |  | 0.15 _ID_ |  |  | 0.15 _ID_ |  |  |
| ICC | 0.05 |  |  | 0.04 |  |  | 0.04 |  |  |
| N | 6 _ID_ |  |  | 6 _ID_ |  |  | 6 _ID_ |  |  |
| Observations | 2282 |  |  | 2282 |  |  | 2282 |  |  |
| Marginal R^2^ / Conditional R^2^ | 0.075 / 0.118 |  |  | 0.077 / 0.118 |  |  | 0.078 / 0.119 |  |  |
| **Low human activity State 2 (Travelling)** | **Top model** | | | **Model 2** | | | **Model 3** | | |
| **Covariates** | **Estimate** | **SE** | **P-value** | **Estimate** | **SE** | **P-value** | **Estimate** | **SE** | **P-value** |
| (Intercept) | -0.35 | 0.14 | 0.013 | -1.43 | 0.17 | <0.001 | -0.34 | 0.12 | 0.007 |
| Dist. Road | -0.07 | 0.04 | 0.138 | -0.13 | 0.06 | 0.028 |  |  |  |
| **Dist. Tea plantation** | **-0.5** | **0.06** | **<0.001** | **-0.58** | **0.09** | **<0.001** | **-0.49** | **0.06** | **<0.001** |
| **Dist. Forest** | **-0.16** | **0.05** | **<0.001** | **-0.38** | **0.07** | **<0.001** | **-0.16** | **0.05** | **<0.001** |
| Dist. River |  |  |  | -0.15 | 0.06 | 0.007 |  |  |  |
| Dist. PA |  |  |  |  |  |  | 0.06 | 0.05 | 0.205 |
| **Random Effects** | | | | | | | | | |
| σ^2^ | 3.29 |  |  | 3.29 |  |  | 3.29 |  |  |
| τ_00_ | 0.11 _ID_ |  |  | 0.15 _ID_ |  |  | 0.08 _ID_ |  |  |
| ICC | 0.03 |  |  | 0.04 |  |  | 0.02 |  |  |
| N | 6 _ID_ |  |  | 6 _ID_ |  |  | 6 _ID_ |  |  |
| Observations | 3247 |  |  | 2282 |  |  | 3247 |  |  |
| Marginal R^2^ / Conditional R^2^ | 0.062 / 0.091 |  |  | 0.077 / 0.118 |  |  | 0.055 / 0.076 |  |  |
| **Moderate human activity State 1 (Resting)** | **Top model** | | | **Model 2** | | | **Model 3** | | |
| **Covariates** | **Estimate** | **SE** | **P-value** | **Estimate** | **SE** | **P-value** | **Estimate** | **SE** | **P-value** |
| (Intercept) | -0.98 | 0.14 | <0.001 | -0.97 | 0.15 | <0.001 | -0.98 | 0.14 | <0.001 |
| **Dist. Road** | **-0.2** | **0.05** | **<0.001** | **-0.21** | **0.05** | **<0.001** | **-0.2** | **0.05** | **<0.001** |
| **Dist. River** | **-0.09** | **0.04** | **0.031** | **-0.1** | **0.04** | **0.021** | **-0.09** | **0.04** | **0.029** |
| **Dist. Settlement** | **0.27** | **0.07** | **<0.001** | **0.24** | **0.07** | **0.001** | **0.27** | **0.07** | **<0.001** |
| **Dist. Tea plantation** | **-0.46** | **0.05** | **<0.001** | **-0.52** | **0.07** | **<0.001** | **-0.46** | **0.06** | **<0.001** |
| Dist. Forest |  |  |  | -0.08 | 0.06 | 0.164 |  |  |  |
| Dist. PA |  |  |  |  |  |  | -0.02 | 0.05 | 0.658 |
| **Random Effects** | | | | | | | | | |
| σ^2^ | 3.29 |  |  | 3.29 |  |  | 3.29 |  |  |
| τ_00_ | 0.09 _ID_ |  |  | 0.11 _ID_ |  |  | 0.09 _ID_ |  |  |
| ICC | 0.03 |  |  | 0.03 |  |  | 0.03 |  |  |
| N | 6 _ID_ |  |  | 6 _ID_ |  |  | 6 _ID_ |  |  |
| Observations | 3116 |  |  | 3116 |  |  | 3116 |  |  |
| Marginal R^2^ / Conditional R^2^ | 0.095 / 0.119 |  |  | 0.099 / 0.127 |  |  | 0.095 / 0.120 |  |  |
| **Moderate human activity State 2 (Travelling)** | **Top Model** | | | **Model 2** | | | **Model 3** | | |
| **Covariates** | **Estimate** | **SE** | **P-value** | **Estimate** | **SE** | **P-value** | **Estimate** | **SE** | **P-value** |
| (Intercept) | -0.46 | 0.13 | <0.001 | -0.47 | 0.14 | 0.001 | -0.46 | 0.14 | 0.001 |
| **Dist. Road** | **-0.2** | **0.04** | **<0.001** | **-0.19** | **0.04** | **<0.001** | **-0.2** | **0.04** | **<0.001** |
| Dist. River | -0.06 | 0.04 | 0.139 |  |  |  | -0.06 | 0.04 | 0.119 |
| Dist. Settlement | 0.08 | 0.04 | 0.052 | 0.07 | 0.04 | 0.074 | 0.07 | 0.04 | 0.126 |
| **Dist. Tea plantation** | **-0.45** | **0.05** | **<0.001** | **-0.44** | **0.05** | **<0.001** | **-0.48** | **0.06** | **<0.001** |
| Dist. Forest |  |  |  |  |  |  | -0.04 | 0.05 | 0.461 |
| **Random Effects** | | | | | | | | | |
| σ^2^ | 3.29 |  |  | 3.29 |  |  | 3.29 |  |  |
| τ_00_ | 0.09 _ID_ |  |  | 0.10 _ID_ |  |  | 0.10 _ID_ |  |  |
| ICC | 0.03 |  |  | 0.03 |  |  | 0.03 |  |  |
| N | 6 _ID_ |  |  | 6 _ID_ |  |  | 6 _ID_ |  |  |
| Observations | 3607 |  |  | 3607 |  |  | 3607 |  |  |
| Marginal R^2^ / Conditional R^2^ | 0.075 / 0.100 |  |  | 0.077 / 0.103 |  |  | 0.078 / 0.105 |  |  |
| **High human activity State 1 (Resting)** | **Top model** | | | **Model 2** | | | **Model 3** | | |
| **Covariates** | **Estimate** | **SE** | **P-value** | **Estimate** | **SE** | **P-value** | **Estimate** | **SE** | **P-value** |
| (Intercept) | -0.9 | 0.22 | <0.001 | -0.9 | 0.21 | <0.001 | -0.91 | 0.21 | <0.001 |
| **Dist. Road** | **-0.17** | **0.05** | **0.001** | **-0.17** | **0.05** | **0.001** | **-0.17** | **0.05** | **0.001** |
| **Dist. River** | **-0.18** | **0.05** | **<0.001** | **-0.19** | **0.05** | **<0.001** | **-0.19** | **0.05** | **<0.001** |
| **Dist. Settlement** | **0.28** | **0.08** | **<0.001** | **0.26** | **0.08** | **0.002** | **0.27** | **0.08** | **0.001** |
| **Dist. Tea plantation** | **-0.44** | **0.06** | **<0.001** | **-0.48** | **0.08** | **<0.001** | **-0.45** | **0.06** | **<0.001** |
| Dist. Forest |  |  |  | -0.05 | 0.07 | 0.505 |  |  |  |
| Dist. PA |  |  |  |  |  |  | -0.03 | 0.05 | 0.555 |
| **Random Effects** | | | | | | | | | |
| σ^2^ | 3.29 |  |  | 3.29 |  |  | 3.29 |  |  |
| τ_00_ | 0.26 _ID_ |  |  | 0.25 _ID_ |  |  | 0.24 _ID_ |  |  |
| ICC | 0.07 |  |  | 0.07 |  |  | 0.07 |  |  |
| N | 6 _ID_ |  |  | 6 _ID_ |  |  | 6 _ID_ |  |  |
| Observations | 2407 |  |  | 2407 |  |  | 2407 |  |  |
| Marginal R^2^ / Conditional R^2^ | 0.080 / 0.146 |  |  | 0.081 / 0.146 |  |  | 0.081 / 0.145 |  |  |
| **High human activity State 2 (Travelling)** | **Top model** | | | **Model 2** | | | **Model 3** | | |
| **Covariates** | **Estimate** | **SE** | **P-value** | **Estimate** | **SE** | **P-value** | **Estimate** | **SE** | **P-value** |
| (Intercept) | -0.37 | 0.11 | 0.001 | -0.37 | 0.11 | 0.001 | -0.37 | 0.11 | 0.001 |
| **Dist. Road** | **-0.11** | **0.05** | **0.018** | **-0.11** | **0.05** | **0.015** | **-0.11** | **0.05** | **0.021** |
| **Dist. River** | **-0.14** | **0.05** | **0.002** | **-0.15** | **0.05** | **0.002** | **-0.14** | **0.05** | **0.002** |
| **Dist. Settlement** | **0.13** | **0.05** | **0.006** | **0.11** | **0.05** | **0.019** | **0.13** | **0.05** | **0.006** |
| **Dist. Tea plantation** | **-0.38** | **0.05** | **<0.001** | **-0.41** | **0.07** | **<0.001** | **-0.38** | **0.05** | **<0.001** |
| Dist. Forest |  |  |  | -0.04 | 0.06 | 0.461 |  |  |  |
| Dist. PA |  |  |  |  |  |  | 0.01 | 0.05 | 0.772 |
| **Random Effects** | | | | | | | | | |
| σ^2^ | 3.29 |  |  | 3.29 |  |  | 3.29 |  |  |
| τ_00_ | 0.26 _ID_ |  |  | 0.25 _ID_ |  |  | 0.24 _ID_ |  |  |
| ICC | 0.07 |  |  | 0.07 |  |  | 0.07 |  |  |
| N | 6 _ID_ |  |  | 6 _ID_ |  |  | 6 _ID_ |  |  |
| Observations | 2407 |  |  | 2407 |  |  | 2407 |  |  |
| Marginal R^2^ / Conditional R^2^ | 0.080 / 0.146 |  |  | 0.081 / 0.146 |  |  | 0.081 / 0.145 |  |  |

**Supplementary Table S8 Major predictor variables considered for resource selection analysis**

| **Serial Number** | **Type of variable** | **Predictor variable** | **Unit** | **Scale** | **Source** |
| --- | --- | --- | --- | --- | --- |
| 1. | Landscape variables | Distance to protected areas | M | NA | Moef&CC, Govt. of India |
| 2. |  | Distance to forests | M | 30 m | Landsat 8 TM |
| 3. |  | Distance to agriculture fields | M | 30 m | Landsat 8TM |
| 4. |  | Distance to tea plantations | M | 30 m | Landsat 8TM |
| 5. | Water sources | Distance to rivers | M | NA | Digital chart of the world, Columbia University |
| 6. | Anthropogenic variables | Distance to human settlements | M | 30 m | Landsat 8TM |
| 7. |  | Distance to roads | M | NA | Roads and Drainage Layer, Digital Chart of the World |

**Supplementary Figure S1 Mean step length (km) with standard error of collared leopards separated by movement state across wet (June-October) and dry (November-May) seasons for leopards tracked with GPS collars in North Bengal, India from 2017-2020. State 1 represents resting locations whereas state 2 represents travelling locations**

**Supplementary Figure S2 Mean turn angle (radians) with standard error of collared leopards separated by movement state across wet (June-October) and dry (November-May) seasons for leopards tracked with GPS collars in North Bengal, India from 2017-2020. State 1 represents resting locations whereas state 2 represents travelling locations**

**Supplementary Figure S3 Mean turn angle (radians) with standard error of collared leopards separated by movement state across low, moderate, and high periods of human activity for leopards tracked with GPS collars in North Bengal, India from 2017-2020. State 1 represents resting locations whereas state 2 represents travelling locations**
